# Severe Acute Respiratory Syndrome Coronavirus 2 (SARS-CoV-2) Mutational Pattern in the Fourth Pandemic Phase in Greece

**DOI:** 10.3390/cimb44010024

**Published:** 2022-01-11

**Authors:** Panagiotis Halvatsiotis, Sofia Vassiliu, Panagiotis Koulouvaris, Kalliopi Chatzantonaki, Konstantinos Asonitis, Ekatherina Charvalos, Argyris Siatelis, Dimitra Houhoula

**Affiliations:** 1 2nd Propaedeutic Department of Internal Medicine, School of Medicine, National and Kapodistrian University of Athens, “ATTIKON” University Hospital, 12461 Chaidari, Greece; 2 School of Medicine, National and Kapodistrian University of Athens, 11527 Goudi, Greece; sofia_vassiliu@hotmail.com; 3 1st Department of Orthopaedics, School of Medicine, National and Kapodistrian University of Athens, “ATTIKON” University Hospital, 12461 Chaidari, Greece; info@drkoulouvaris.gr; 4 Invitrolabs SA Leventi 9, 12132 Attiki, Greece; popixatz@hotmail.com (K.C.); directorcentralabs@iaso.gr (E.C.); 5 Division of Hematology and Central Hematology Laboratory, Lausanne, Department of Hematology University Hospital (CHUV) and University of Lausanne (UNIL), 1011 Lausanne, Switzerland; k.asonitis@icloud.com; 6 3rd Department of Urologist, School of Medicine, National and Kapodistrian University of Athens, “ATTIKON” University Hospital, 12461 Chaidari, Greece; argysiat@yahoo.gr; 7 Department of Food Science and Technology, University of West Attica, Agiou Spyridonos 28, 12243 Aigaleo, Greece; dhouhoula@uniwa.gr

**Keywords:** RT-PCR, *S* gene, COVID-19, SARS-CoV-2, Greece, variants, mutations

## Abstract

The aim of this study is to investigate the circulating variants of severe acute respiratory syndrome coronavirus 2 (SARS-CoV-2) from Athens and from rural areas in Greece during July and August 2021. We also present a rapid review of literature regarding significant SARS-CoV-2 mutations and their impact on public health. A total of 2500 nasopharyngeal swab specimens were collected from suspected COVID-19 cases (definition by WHO 2021b). Viral nucleic acid extraction was implemented using an automatic extractor and the RNA recovered underwent qRT-PCR in order to characterize the specimens as positive or negative for SARS-CoV-2. The positive specimens were then used to identify specific Spike gene mutations and characterize the emerging SARS-CoV-2 variants. For this step, various kits were utilized. From the 2500 clinical specimens, 220 were tested positive for SARS-CoV-2 indicating a prevalence of 8.8% among suspected cases. The RT-PCR Ct (Cycle threshold) Value ranged from 19 to 25 which corresponds to medium to high copy numbers of the virus in the positive samples. From the 220 positive specimens 148 (67.3%) were from Athens and 72 (32.7%) from Greek rural areas. As far as the Spike mutations investigated: N501Y appeared in all the samples, D614G mutation appeared in 212 (96.4%) samples with a prevalence of 87.2% in Athens and 98.6% in the countryside, E484K had a prevalence of 10.8% and 12.5% in Athens and the rural areas, respectively. K417N was found in 18 (12.2%) samples from Athens and four (5.6%) from the countryside, P681H was present in 51 (34.5%) Athenian specimens and 14 (19.4%) specimens from rural areas, HV69-70 was carried in 32.4% and 19.4% of the samples from Athens and the countryside, respectively. P681R had a prevalence of 87.2% in Athens and 98.6% in rural areas, and none of the specimens carried the L452R mutation. 62 (28.2%) samples carried the N501Y, P681H, D614G and HV69-70 mutations simultaneously and the corresponding variant was characterized as the Alpha (UK) variant (B 1.1.7). Only six (2.7%) samples from the center of Athens had the N501Y, E484K, K417N and D614G mutations simultaneously and the virus responsible was characterized as the Beta (South African) variant (B 1.351). Our study explored the SARS-CoV-2 variants using RT-PCR in a representative cohort of samples collected from Greece in July and August 2021. The prevalent mutations identified were N501Y (100%), D614G (96.4%), P681R (90.1%) and the variants identified were the Delta (90.1%), Alpha (28.2%) and Beta (2.7%).

## 1. Introduction

In December 2019 many cases of pneumonia without a defined causative factor were reported in Wuhan China, with some patients rapidly developing respiratory distress syndrome or acute respiratory failure [1]. On 7 January a novel coronavirus was identified by the Chinese Center for Disease Control and Prevention (CCDC) from the throat swab sample of a patient and was subsequently named ‘2019-nCoV’ by the WHO [2]. The 2019-nCoV infection caused clusters of severe respiratory illness similar to severe acute respiratory syndrome. Most patients were men and a sizable proportion suffered from underlying conditions [3]. The first COVID-19 case in Greece was diagnosed on 26 February of 2020 and on March 23rd with 695 confirmed cases and 17 deaths a nation-wide restriction on freedom of movement was enforced. On 8 March the first patient with confirmed pneumonia by 2019-nCoV was admitted in an Intensive Care Unit (ICU) due to pulmonary failure. Globally, up until 29 October 2021, there have been 245,373,039 confirmed cases of COVID-19, including 4,979,421 deaths, reported to WHO [4]. In Greece, for the same time period, a total number of 734,778 cases have been reported with 15,856 registered deaths [5]. The virus continued to devastate public health and economy worldwide enduring a second, a third and now a fourth wave of outbreaks, and only mass vaccination could protect the general public. A high vaccination rate is essential in order to approach herd immunity and to bring the pandemic under control (Mathieu et al., 2021). Up to 28 October 2021, a total of 6,838,727,352 vaccine doses have been administered worldwide [4] and 12,692,458 in Greece [6].

SARS-CoV-2, like other RNA viruses, is prone to genetic evolution with the development of mutations over time, resulting in the emergence of multiple variants that may have different characteristics compared to its ancestral strains. New variants may possess the ability to evade available detection methods, therapeutic regimens and reduce vaccination effectiveness. Even a single mutation can drastically affect a virus’ ability to evade the immune system and complicate the vaccine development progress against the virus. Adaptive mutations in the viral genome can alter the virus’s pathogenic potential and the continuous emergence of new virus variants with increased infectivity have obliged the scientific community to be vigilant for the virus evolution. Multiple variants of SARS-CoV-2 have been described, of which a few are considered variants of concern (VOCs), given their impact on public health [7]. Thus, to effectively control the pandemic, it is considered crucial to monitor the possible emergence of rare mutations especially in the spike-encoding region [8]. Tracking the SARS-CoV-2 variants and their mutational patterns in different areas not only can assist in the implementation of appropriate measures to protect the population (e.g., from super spreaders) but also provides valuable data to the vaccine development platform [7,9]. This study aims to investigate SARS-CoV-2 mutations in various regions of Greece.

## 2. Materials and Methods

### 2.1. Clinical Samples

A total of 2500 clinical specimens were collected from the center of Athens and from rural areas in Greece during July and August 2021. All volunteers met the suspected COVID-19 case definition set by the World Health Organization (World Health Organization, 2021b). Sampling was accomplished with all the required precautions. Nasopharyngeal swabs were collected and subsequently placed in 2 mL of transport medium with neutralizing agent. Disposable Virus Sampling Tube (Zybio Inc.; Chongqing, China) adopts efficient virus inactivation technology and special flocked swab. It can be used for the collection and storage of clinical novel coronavirus, influenza, avian influenza (such as H7N9), hand-foot-mouth virus, measles and other virus specimens, as well as chlamydia, mycoplasma, and ureaplasma. Specimen processing was performed in a class II biological safety cabinet using biosafety level three (BSL3) work practices.

### 2.2. RNA Extraction

Nucleic acids were recovered from clinical specimens using an automatic extractor (MagDEA DNA/RNA 200 virus). The RNA extracted from each specimen was divided in two aliquots and stored at −20 °C for the experiments. 

### 2.3. qRT PCR

The first aliquot was used for the identification of the SARS-CoV-2 positive specimens through qRT-PCR. The Mutaplex SARS-CoV-2 commercial kit (Immundiagnostik AG) was utilized for this purpose. Specific primers were used for highly conserved regions and double-labeled probes to enhance and differentiate RNA SARS-CoV-2 from other beta Coronaviridae such as MERS. Detection of SARS-CoV-2 was visualized at the FAM/GREEN channel. Betacoronaviruses (SARS-CoV-1 and SARS-CoV-2) are detected at Cy5/RED channel. Internal Process Control (IPC), which was added during RNA extraction, was detected in the same reaction at HEX/YELLOW. Detection of RNA Polymerase (human gene) allows RT-PCR detection of inhibitors confirming in addition viral RNA was isolated from specimen. 

### 2.4. Typing qRT PCR Kits

For this, the RNA used derived from the second aliquots of the 220 SARS-CoV-2-positive specimens. Different kits were utilized to identify the mutations and characterize the variants according to the manual procedures.

PhoenixDx^®^ SARS-CoV-2 Mutant Screen for [D614G], [N501Y], [del HV69/70], [E484K], [K417N], [P681H], [P681R] and [L452R] are separate real-time RT-PCR-based diagnostic tests for in vitro discrimination between wildtype SARS-CoV-2 and the respective SARS-CoV-2 mutants.

GeneProof SARS-CoV2 South African (B 1.351)/UK (B 1.1.7)/Brazilian (P 1) typing PCR Kit is a qualitative real-time PCR that identifies the three variants -B 1.135, B 1.1.7 and P 1- through targeting specific sequences of the SARS-CoV-2 genome (*RdRp* and *S* gene). The presence of SARS-CoV2 is indicated by the increased fluorescence in FAM Channel, the UK variant in Cy5 channel, South African variant in HEX Channel and Brazilian variant in Texas Red Channel. 

Apart from the use of the aforementioned kit, the different variants were also characterized by the combination of mutations they held according to WHO. The results of the two different methods are described below.

## 3. Results

From the 2500 RNA specimens, 220 were tested positive for SARS-CoV-2 indicating a prevalence of 8.8% among suspicious cases. The RT-PCR Ct Value ranged from 19 to 25 that corresponds to medium to high copy numbers of the virus in the positive specimens of our study. From the 220 positive samples 148 (67.3%) were from the center of Athens and 72 (32.7%) from rural areas throughout Greece.

The N501Y spike mutation has been identified in three variants of concern: Alpha, Beta and Gamma. This mutation causes greater affinity of the variants for human ACE2 (Angiotensin-Converting Enzyme 2), which constitutes the receptor on host cell surfaces though which SARS-CoV-2 can enter and start replicating [10,11]. All the 220 (100%) positive samples in our study had the N501Y mutation. D614G mutation constitutes an amino acid substitution in the spike protein which was identified early in the course of the pandemic and has now become the prevalent mutation of SARS-CoV-2 globally [12]. It is carried by the majority of circulating variants and does not increase the risk of hospitalization or have an impact to the antibody binding to the S protein [13]. However, in animal and in vitro studies, it has been connected with higher levels of infectious virus in the respiratory tract, enhanced binding to ACE-2, and increased replication and transmissibility [14,15]. In our study, 212 (96.4%) samples were positive for the D614G Spike mutation, with a prevalence of 87.2% and 98.6% in Athens and rural areas, respectively. Lower prevalence was found to the E484K Spike Mutation; 10.8% in Athens and 12.5% in the countryside. It has been suggested that this mutation impacts immunity -either natural or from vaccination- and may thus also reduce the effectiveness of treatment with monoclonal antibodies, convalescent plasma and can even cause reinfection to certain patients, mostly immunocompromised [16,17,18].

The K417N/T mutation is located on the border of the receptor binding motif (RBM) [19], the part of the receptor binding domain (RBD) that mediates contact with ACE2 [20]. From the 148 positive samples collected from Athens 18 (12.2%) had this mutation contrary to the samples from rural areas in which four out of 72 (5.6%) had it. The P681H mutation, which is considered to have an exponential increase in global frequency [21] in our study was identified in 51 (34.5%) and 14 (19.4%) samples from Athens and rural areas, respectively. The HV69-70del mutation is a deletion at amino acid positions 69 and 70 of the spike protein and leads to certain molecular tests’ inability to detect the *S* gene (encoding the spike protein) but no false negative results since these tests target more than one gene. Thus, *S* gene target failure has been utilized to detect the Alpha variant, which does not carry this deletion [22]. The prevalence of this mutation in our study was found 32.4% and 19.4% in Athens and in the Greek countryside, respectively. P681R spike mutation is considered to be responsible for the increased infectivity of the Delta variant, to a significant degree [23]. The P681R spike mutation increases the replication of the Delta variant by increasing the dissociation of S1 and S2 subunits at the furin cleavage site [24]. In this study the P681R mutation had a prevalence of 87.2 and 98.6% in Athens and rural areas, respectively. None of the samples carried the L452R mutation. The prevalence of the various mutations in our study is presented in Table 1.

Each variant carries a specific combination of mutations. In our study, the 62 (28.2%) samples that had the N501Y, P681H, D614G and HV69-70 mutations simultaneously were characterized as the UK (B 1.1.7) variant. Only 6 (2.7%) samples from the center of Athens had the N501Y, E484K, K417N and D614G mutations simultaneously and were characterized as South African (B 1.351) variants. In both the above cases, the same results were found using the Gene Proof kit. The prevalence of the identified variants in our study as well as more information on the VOCs is presented in Table 2.

## 4. Discussion

Our study explored the SARS-CoV-2 mutational pattern in a representative cohort of samples collected from the center of Athens and several rural areas around Greece covering a period from early July till the end of August 2021. We chose to investigate certain key mutations which, according to current literature, have an important impact on public health or are carried by SARS-CoV-2 variants of concern. The prevalent mutations that we identified were the following spike mutations: N501Y (100%), D614G (96.4%) and P681R (90.1%) and the emerging variants were the Delta (90.1%), Alpha (28.2%) and Beta (2.7%).

The RT-qPCR technique was implemented for the identification of the mutations and variants since it is more affordable than other molecular techniques, such as sequencing. We suggest that this screening strategy could therefore be implemented more easily in most countries. Undoubtedly, if sequencing is also an option, it offers not only confirmation of the RT-PCR findings but also epidemiological advantages through the identification of both known and novel transcripts. No confirmation of our findings by sequencing and the relatively restricted sample of specimens used constitute the limitations of our study. As far as the former is concerned, although it does represent a weakness for analyzing clinical samples, it is not a strong disadvantage considering the main objective of these assays, which is to analyze samples more conveniently, in terms of money and time to keep molecular epidemiological surveillance in near-real-time. Therefore, we believe that these assays could provide information that helps prevent and control future outbreaks generated by the introduction of variants in new geographical locations [25]. 

Different designations have been given to the variants by different phylogenetic classification systems (e.g., Pango lineage, Nextstrain, GISAID). The WHO uses letters of the Greek alphabet to name distinct variants and has also categorizes the variants based on their properties and epidemiological impact [7]. Therefore, the Alpha, Beta, Gamma and Delta variants are considered variants of concern (VOC) according to WHO due to their important impact on transmissibility, severity and immunity affecting public health globally. Interestingly, the ECDC -with a similar classification system of the variants- classifies the Alpha not as a VOC but as a “De-escalated Variant”: either no longer circulating or with no impact and concerning properties [8]. The prevalence of the Alpha variant has declined worldwide after the emergence of the Delta variant [26].

Finding mutations, especially with known clinical impact, might have significant implications for public health policies, surveillance and immunization strategies; therefore, we consider that the world’s health authorities should promote tracking of mutations with important biological effects even through rapid screening techniques [17,27]. The fact that this pandemic has beset health, healthcare systems and the economy worldwide dictates the urgency for affordable and rapid techniques for SARS-CoV-2 mutations’ and variants’ immediate and effective global surveillance.

In conclusion, this manuscript describes the application of RT-qPCR assays to detect some mutations of interest in Greece at this pandemic period. We investigated three mutations of concern in the collected samples. SARS-CoV-2′s mutational pattern was explored through rapid molecular screening and is reported here for the first time in Greece, to our knowledge.

## Figures and Tables

**Table 1:** Spike mutations investigated in our study and their proportion in the collected specimens.

Spike Mutation	(+) Specimens from Athens (%)	(+) Specimens from Greek Rural Areas (%)	(+) Specimens in Total (%)
N501Y	148 (100)	72 (100)	220 (100)
D614G	129 (87.2)	71 (98.6)	212 (96.4)
E484K	16 (10.8)	9 (12.5)	25 (11.3)
K417N/T	18 (12.2)	4 (5.6)	58 (26.3)
HV69-70DEL	48 (32.4)	14 (19.4)	62 (28.1)
P681R	129 (87.2)	71 (98.6)	200 (90.1)
P681H	51 (34.5)	14 (19.4)	65 (29.5)
L452R	0 (0)	0 (0)	0 (0)

**Table 2:** SARS-CoV-2 variants and their proportion in the collected specimens of our study.

WHO Label	Pango Lineage	Spike Mutations	Country First Detected	(+) Specimens from Athens (%)	(+) Specimens from Greek Rural Areas (%)
Alpha	B.1.1.7	N501Y D614G P681H HV69-70del	UNITED KINGDOM	49 (33.1%)	13 (18.1%)
Beta	B.1.135	K417N E484K N501Y D614G	SOUTH AFRICA	6 (4.1%)	0 (0%)
Gamma	P.1	K417T E484K N501Y D614G	BRAZIL	0 (0%)	0 (0%)
Delta	B.1.617.2	D614G P681R	INDIA	129 (87.2%)	71 (98.6%)

## Data Availability

The data presented in this study are available on request from the corresponding author.
